# Photo‐ and Electrochemical Dual‐Responsive Iridium Probe for Saccharide Detection

**DOI:** 10.1002/chem.202103541

**Published:** 2021-12-08

**Authors:** Andrew J. Carrod, Francesco Graglia, Louise Male, Cécile Le Duff, Peter Simpson, Mohamed Elsherif, Zubair Ahmed, Haider Butt, Guang‐Xi Xu, Kenneth Kam‐Wing Lo, Paolo Bertoncello, Zoe Pikramenou

**Affiliations:** ^1^ School of Chemistry University of Birmingham Edgbaston Birmingham B15 2TT UK; ^2^ College of Engineering Swansea University Swansea SA1 8EN UK; ^3^ School of Engineering University of Birmingham Edgbaston Birmingham B15 2TT UK; ^4^ College of Medical and Dental Sciences University of Birmingham Edgbaston Birmingham B15 2TT UK; ^5^ Department of Chemistry City University of Hong Kong Tat Chee Avenue Hong Kong China

**Keywords:** electro-chemiluminescence, iridium, luminescence, probes, saccharides, sensors

## Abstract

Dual detection systems are of interest for rapid, accurate data collection in sensing systems and in vitro testing. We introduce an Ir^III^ complex with a boronic acid receptor site attached to the 2‐phenylpyridine ligand as an ideal probe with photo‐ and electrochemical signals that is sensitive to monosaccharide binding in aqueous solution. The complex displays orange luminescence at 618 nm, which is reduced by 70 and 40 % upon binding of fructose and glucose, respectively. The electro‐chemiluminescent signal of the complex also shows a direct response to monosaccharide binding. The Ir^III^ complex shows the same response upon incorporation into hydrogel matrices as in solution, thus demonstrating the potential of its integration into a device, as a nontoxic, simple‐to‐use tool to observe sugar binding over physiologically relevant pH ranges and saccharide concentrations. Moreover, the complex's luminescence is responsive to monosaccharide presence in cancer cells.

## Graphical abstract

**Multimodal sensing**: A luminescent iridium(III) complex, designed with boronic acid groups closely coupled to the metal centre, responds to sugar binding upon optical and electrochemical stimuli. The orange iridium luminescence signal is quenched by sugar binding in aqueous solutions at physiological pH. The signal is also observed in cancer cells, and is quenched by sugars at concentrations mimicking blood plasma. Hydrogel matrices with incorporated iridium complex retain the sensing response to sugars, providing an exemplar transition metal complex for saccharide‐responsive devices.

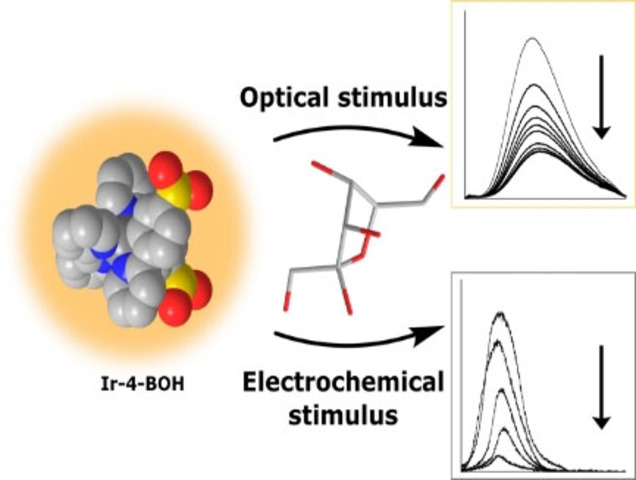

## Introduction

**Scheme 1:**
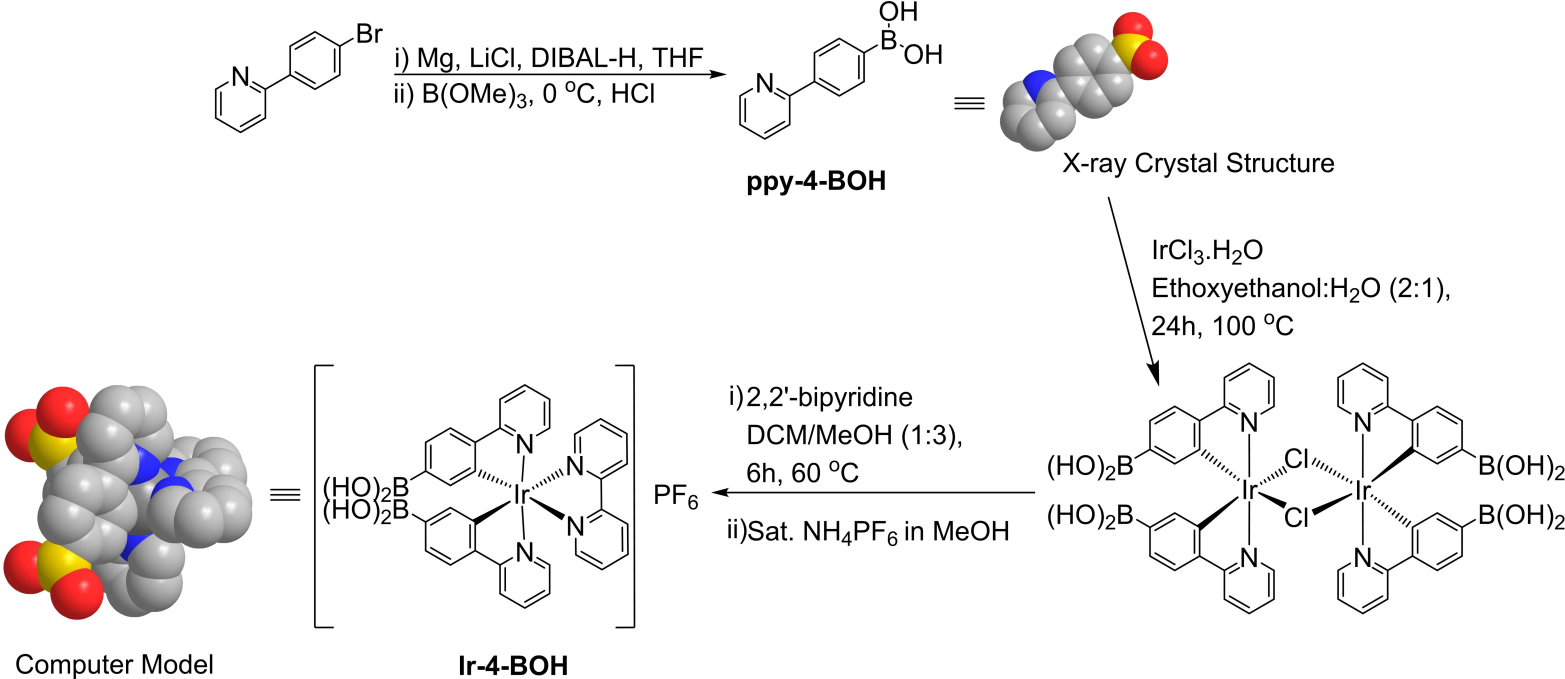
Scheme for the synthesis of the Ir‐4‐BOH complex.

Dual‐modality sensing by optical and electrochemical techniques is a popular detection strategy for analytes, not only for the high sensitivity but also as an approach to eliminate false positives due to the differing signal transduction pathways in each detection method.^[1]^ Despite the demonstrated versatile integration of optoelectronic probes in portable chip devices, and several reports of dual sensors for other analytes, the development of multimodal sensing for sugars is relatively unexplored.^[2]^ Supramolecular recognition of *cis*‐1,2 and 1,3 diols by using boronic acid receptors is a popular approach,^[3]^ based on either the detection of fluorescence signal arising from organic probes,^[4]^ or electrochemical responses of organic compounds and ferrocene metal complexes.^[5]^ It is a challenge to develop water soluble compounds with dual photo‐ and electrochemical response while retaining optimized properties for saccharide binding. Transition metal complexes offer dual‐mode electrochemical and photoluminescence signals.^[6]^ The design of polypyridine luminescent transition metal complexes with boronic acid recognition groups has been limited to the boronic acid connected either via a long aliphatic linker to the oligopyridine unit, or via phenyl linker. In these cases either weak or no luminescent signal response was observed upon saccharide binding; alternatively conditions of high pH beyond physiological range were necessary to illicit a response. Furthermore, the studies were based on a single detection method.^[7]^ We chose an iridium(III) photoactive core based on the sensitivity of the luminescence properties to local environmental changes, but also for electrochemical properties.^[8]^ Herein, we report an iridium(III) complex, Ir‐4‐BOH, with two boronic acid recognition sites grafted on the phenylpyridine ligand to bring the recognition site spatially closer to the metal centre. Using this cyclometallated ligand. the saccharide binding event should influence both lower metal charge transfer excited states and redox properties, in order to trigger dual electrochemical and luminescence signal responses (Scheme 1). The versatility in the design of iridium(III) complexes as sensors can overcome challenges from solubility, working pH range, detection in different areas of the spectrum whilst providing a framework to build a recognition site.

## Results and Discussion

The complex Ir‐4‐BOH was synthesized based on adapted procedures^[9]^ and a control compound without the boronic acid recognition site, Ir‐ppy, was also prepared and characterized (see the Supporting Information).^[10]^ Single crystals of the ligand ppy‐4‐BOH were obtained by slow evaporation of a solution in MeOH, the X‐ray crystal structure and selected data are displayed in Figure S1 in the Supporting Information along with experimental considerations.

The photophysical properties of Ir‐4‐BOH were compared with those of Ir‐ppy in both CH_3_CN and aqueous solutions (0.01 M PBS with 2 % CH_3_CN; Tables 1 and S1). The UV‐vis absorption spectrum of Ir‐4‐BOH (Figure S2) displays peaks at 260, 350 and 400 nm consistent with previous reports of ligand‐centred and charge transfer transitions.[^10^, ^11^] An aqueous solution of the complex displays an emission band centred at 618 nm. The overall photophysical characterization data show similar excited state behaviour with the Ir‐ppy, with slightly shorter luminescence lifetimes and lower quantum yields, which is expected based on the weak electron withdrawing effect of the B(OH)_2_ substituent.

**Table 1:** Selected photophysical data for Ir‐4‐BOH in aqueous ([PBS]=0.01 M, 2 % CH_3_CN) and acetonitrile solutions. Estimated errors Δ*λ* ±1 nm, Δ*τ* ±10 %, emission quantum yield *Φ* ±20 % of given value.

Solvent	*λ*_max_ [nm]	*Φ* [%] aerated (deaerated)	*τ* [ns] aerated (deaerated)
water	618	0.4 (1.4)	40 (45)
CH_3_CN	610	4.2 (12)	55 (320)

We chose to investigate the Ir‐4‐BOH complex as a probe for glucose and fructose concentration in aqueous solutions. Glucose is well known for its role in detection of diabetes mellitus and dominates in concentration in human plasma by at least two orders of magnitude in comparison to other sugars. Fructose is found at far lower concentrations in human blood, however it has been shown that it binds stronger than glucose to boronic acid receptors. This is due to the higher availability of the β‐d‐fructofuranose form than the α‐d‐glucofuranose, with each of these forms bearing a *syn*‐periplanar pair of hydroxyl groups available for boronic acid binding.^[4c]^ Therefore, fructose can potentially act as an interferent in any glucose assay.

We examined the binding of glucose and fructose to Ir‐4‐BOH by titrations in aqueous solutions. The UV‐vis spectrum of the complex was monitored upon each addition of the monosaccharide (Figure S3), the absorption peak centred at 260 nm decreased in intensity, with a total reduction of 18 % for glucose and 15 % for fructose, at [monosaccharide]=50 mM. The charge transfer bands at 350 and 400 nm do not show significant changes. The same experiment was performed using the control complex Ir‐ppy and no reduction in the intensity of any of the absorption bands was observed (Figure S4).

**Figure 1:**
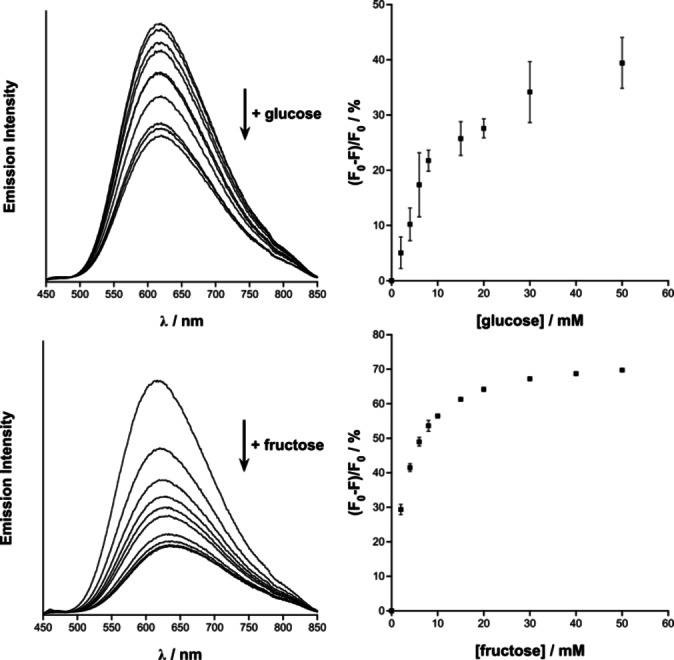
Left: Emission spectra of Ir‐4‐BOH (40 μM in water with 0.01 M PBS and 2 % CH_3_CN, pH 7.4) upon additions of glucose (top) and fructose (bottom). Monosaccharide concentrations 0, 2, 4, 6, 8, 10, 20, 30, 40 and 50 mM. Right: Fitted percentage decrease plots for glucose (top) and fructose (bottom) are also displayed with error bars shown as the standard deviation, *N*=3.

Monitoring of the luminescence spectra of Ir‐4‐BOH in aqueous solutions with increasing concentrations of monosaccharide shows a significant change in the ^3^MLCT band. Upon addition of aliquots of the monosaccharide to a solution of Ir‐4‐BOH a decrease in the luminescence signal is observed (Figure 1), up to 40 and 70 % for glucose and fructose, respectively. Upon addition of monosaccharide, no detectable decrease in the luminescence signal was observed for Ir‐ppy (Figure S5), which supports that the change in the luminescence intensity of Ir‐4‐BOH is attributed to the monosaccharide binding to the boronic acid moiety. The relevant changes of the luminescence signal at different saccharide concentrations provide information of the binding of the monosaccharide to the iridium(III) complex, allowing determination of the 1 : 1 binding constants. These were evaluated to be 82±10 and 331±25 M^−1^ for glucose and fructose, respectively (Figure S6) which are two‐fold higher than some recently reported examples of phenylboronic acids and anthracene boronic acids.^[12]^ Interestingly, the binding constants do not follow the trend observed in the case of the previously reported Pt(II) complexes,^[7c]^ possibly due to the charge or the spatial orientation of the boronic acid. However, blood glucose levels lie in the range of 2 to 30 mM,^[13]^ and the Ir‐4‐BOH probe displayed luminescence changes that are detected within the region for biological saccharide monitoring.

**Figure 2:**
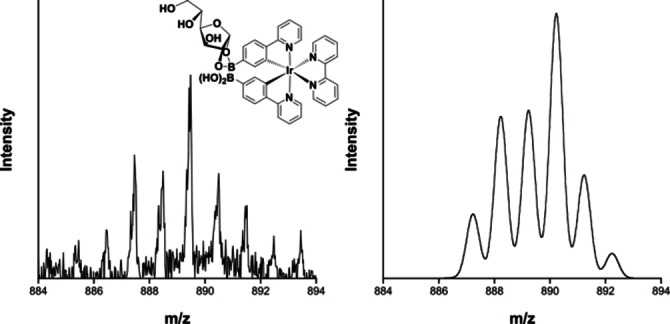
MALDI mass spectrum of the Ir‐4‐BOH**⋅**glucose species and theoretical isotope pattern.

Mass spectrometry was used to identify the species formed upon addition of the monosaccharide to the Ir^III^ complex. A peak centred at *m*/*z* 889 in the MALDI mass spectrum of Ir‐4‐BOH with glucose corresponds to 1 : 1 Ir‐4‐BOH**⋅**glucose species (Figure 2). A much weaker peak is also observed at *m*/*z* 1033 (Figure S7), which corresponds to Ir‐4‐BOH**⋅**(glucose)_2_. Previous studies have confirmed that α‐d‐glucofuranose are the active species for binding.^[14]^ ^11^B and ^19^F NMR spectroscopy was also employed to ratify the presence of the monosaccharide bound species, and comparison with organic boronic acid compounds.[^4b^, ^15^] We monitored the ^11^B and ^19^F signals using active reporter diols 3‐fluorocatechol and 2‐fluoro‐2‐deoxy‐D‐glucose (FDG). A solution of the complex Ir‐4‐BOH with 3‐fluorocatechol shows an additional ligand ^11^B signal at 14 ppm which is indicative of the formation of the boronate ester, also observed in the ligand ppy‐4‐BOH under similar conditions (Figure S8).

This is in agreement with previously reported boronic acid compounds^[16]^ Upon addition of Ir‐4‐BOH the ^19^F NMR spectrum of 3‐fluorocatechol showed an additional peak at −144 ppm, shifted from −138 ppm (Figure S9) and the spectrum of 2‐fluoro‐2‐deoxy‐d‐glucose showed the presence of an additional peak at 198 ppm (Figure S10). The additional peaks confirm the presence of the iridium‐saccharide conjugates, with expected magnitude of shifts.[^15b^, ^17^]

**Figure 3:**
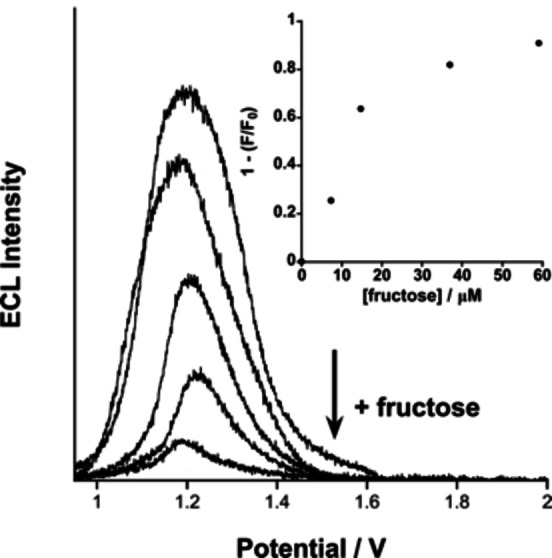
ECL spectra of complex Ir‐4‐BOH with increasing concentrations of fructose, the supporting electrolyte was 0.1 M aqueous NaCl at pH 7.5 and the scan rate 50 mV s‐1. Inset: 1−(*F*/*F*_o_) for each spectrum vs. concentration of fructose.

To take advantage of the electrochemiluminescence (ECL) properties of Ir‐4‐BOH, we examined the effect of monosaccharide on the electrochemiluminescence signal from the Ir^III^ complex as a direct reporter. Most commonly, ECL measurements to sense glucose have been carried out as an indirect method, by changing the properties of solely the co‐reactant and not of the reporter.^[5b]^ The ECL spectra of both Ir‐4‐BOH and Ir‐ppy at pH 7.5 showed an ECL signal at +1.2 V. In the case of Ir‐4‐BOH the observed ECL signal is suppressed by addition of fructose to the electrochemical cell, similarly to what has been observed in our photoluminescence studies (Figure 3).

At a concentration of 60 μM of fructose, the ECL signal intensity is decreased by 90 %. In the control experiment, the ECL of Ir‐ppy shows no correlation to the concentration of fructose.

The electrochemical properties of the complexes are also investigated by cyclic voltammetry (Table 2). The oxidation at +1.19 V for Ir‐4‐BOH is assigned to the Ir^IV/III^ couple. This oxidation peak is significantly shifted from the +1.27 V of the complex without this group Ir‐ppy. Reduction of the bpy ligand occurs at −1.37 and −1.40 V for Ir‐4‐BOH and Ir‐ppy, respectively, which aligns well with reported values for other Ir^III^ complexes.[^11a^, ^18^] We therefore postulate that, due to the lack of functionalisation, the reduction of the bpy ligand is unaffected by any electronic effect of the boronic acid.

**Table 2:** Electrochemical data for complexes Ir‐4‐BOH and Ir‐ppy, taken at 298 K and pH 7.5. Recorded in a 0.1 M aqueous NaCl solution at scan rate of 50 mV/s, referenced to Ag/AgCl electrode.

Complex	Oxidation *E*_1/2_ or *E*_a_ [V]	Reduction *E*_1/2_ or *E*_c_ [V]
Ir‐4‐BOH	+1.19	−1.37
Ir‐ppy	+1.27	−1.40

**Figure 4:**
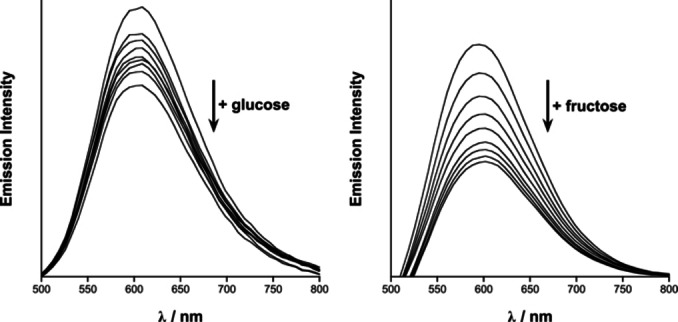
Emission spectra of hydrogel devices with Ir‐4‐BOH immersed in aqueous solution (0.01 M PBS) upon addition of 0, 2, 4, 6, 8, 10, 25, 50 and 100 mM monosaccharide (*λ*_ex_=450 nm).

To examine the suitability of Ir‐4‐BOH for a sensing device, we used hydrogels which are promising platforms for implants, offering applications for continuously monitoring glucose levels and are compatible with organic probes.^[19]^ The luminescence signal of the Ir‐4‐BOH complex impregnated in hydroxyethylmethacrylate hydrogels was monitored. A decrease in intensity upon each subsequent addition of a solution of the monosaccharide was observed (Figure 4).

The gel is responsive to the monosaccharide at low concentrations 2–40 mM, which is ideal for the detection of blood glucose levels. The 1 : 1 binding constants from the interaction in the hydrogel were estimated to be 185±55 and 210±25 M^−1^ (Figures S11 and S12), for glucose and fructose, respectively. The value for glucose is higher than the one observed in solution, which may be attributed to the stronger glucose interaction due to immobilisation of the metal complex in the hydrogel. As a control experiment Ir‐ppy was tested in the hydrogel under the same conditions; no reduction in the luminescence signal was observed when the saccharides were added (Figure S13). For medical application the gel devices must also be non‐toxic and stable. To probe the stability across a 48‐hour window, the emission intensity of the hydrogels was used as an indicator for the release of Ir‐4‐BOH into solution from such a gel device. The devices were immersed in an aqueous solution of 0.01 M PBS and the solution was removed before each measurement to eliminate any emission signal from released Ir‐4‐BOH complex. No signal decrease was observed within 48 h (Figure S14), which indicated that the complex is entrapped, and minimal release occurred at physiological pH. Cytotoxicity testing was carried out over a 72‐hour period on several different cell lines found within human skin; human dermal fibroblasts, keratinocytes, and melanocytes. No toxicity was observed (Figure S15), and it can be concluded that the gels are non‐cytotoxic and would have compatibility as a skin worn or sub‐dermal device.

**Figure 5:**
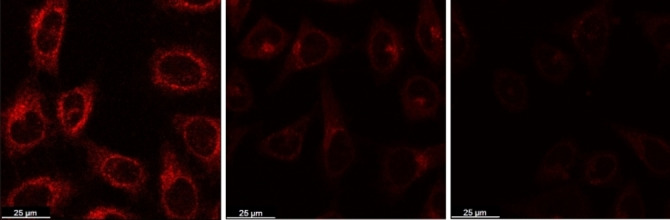
Confocal microscopy images of live HeLa cells stained by Ir‐4‐BOH (10 μM) for 2 h after incubation in a sugar‐free medium (left), a medium containing 50 mM glucose (middle) or fructose (right) at 37 °C for 24 h. Scale bar: 25 μm.

Many cellular pathways important for diagnosing deficiencies involve glucose processing. To examine the potential of the molecular complex as a probe in cellular environments, we examined the Ir‐4‐BOH uptake in cancer cells, by ICP‐OES and confocal microscopy. HeLa cells were incubated for 24 h in either sugar‐free medium, or medium containing glucose or fructose (50 mM) in conditions mimicking low and high concentration of the sugar in blood plasma. The results (Table S2) show that the uptake of the Ir^III^ complex in monosaccharide‐containing media is almost halved. This is ascribed to the decreased lipophilicity of the iridium‐saccharide conjugate. Due to the more efficient uptake, the IC_50_ value of the complex in a sugar free medium (8.0 μM) was much lower than the cases when the medium contained 50 mM glucose (34.7 μM) or fructose (19.1 μM), much higher than the minimum concentration of Ir‐4‐BOH required to detect luminescence changes in saccharide concentration, confirming the suitability of the probe. Confocal microscopy of live HeLa cells revealed a visible reduction in the intensity of the intracellular emission arising from the Ir‐4‐BOH when incubated in media containing saccharide (50 mM; Figure 5). This is due to both the lower cellular uptake efficiencies of the conjugates and the reduced emission intensities of the sugar conjugates compared to the free complex.

## Conclusion

We have demonstrated that the luminescence of an Ir^III^ complex can be used to monitor and detect saccharide binding by using a boronic receptor site close to the iridium metal centre. The design is novel for both electrochemical and photoluminescence responses, which provides a paradigm for coordination‐complex‐based saccharide sensors. This sensor shows large luminescence changes in aqueous solution upon complexation of glucose and fructose. The iridium luminescence allows detection in cells and is responsive to various concentrations of the monosaccharide. Further to this, we have shown that the complex can be used to build saccharide‐sensing hydrogel devices, which provide flexibility, stability, reproducibility and affordability, meaning it could be a useful diagnostic tool for saccharide sensing.

## Additional Information

Deposition Number CCDC 2027423 (for ppy‐4‐BOH) contains the supplementary crystallographic data for this paper. These data are provided free of charge by the joint Cambridge Crystallographic Data Centre and Fachinformationszentrum Karlsruhe Access Structures service.

## Conflict of interest

The authors declare no conflict of interest.

## Supporting information

As a service to our authors and readers, this journal provides supporting information supplied by the authors. Such materials are peer reviewed and may be re‐organized for online delivery, but are not copy‐edited or typeset. Technical support issues arising from supporting information (other than missing files) should be addressed to the authors.

Supporting Information
