# Supplementary material for: Photo‐ and Electrochemical Dual‐Responsive Iridium Probe for Saccharide Detection
Source: Chemistry. 2021 Dec 8;28(4):e202103541. doi: 10.1002/chem.202103541 (PMC9299874; doi:10.1002/chem.202103541)
Supplement: Supplementary file 1 — Supporting Information [file CHEM-28-0-s001.pdf]

Chemistry–A European Journal

Supporting Information

Photo- and Electrochemical Dual-Responsive Iridium Probe for Saccharide Detection

Andrew J. Carrod, Francesco Graglia, Louise Male, Cécile Le Duff, Peter Simpson, Mohamed Elsherif, Zubair Ahmed, Haider Butt, Guang-Xi Xu, Kenneth Kam-Wing Lo, Paolo Bertoncello, and Zoe Pikramenou*

Table of Contents

Supporting Figures and Tables p 2

NMR Spectra p 10

Experimental Section, Materials and Methods p 13

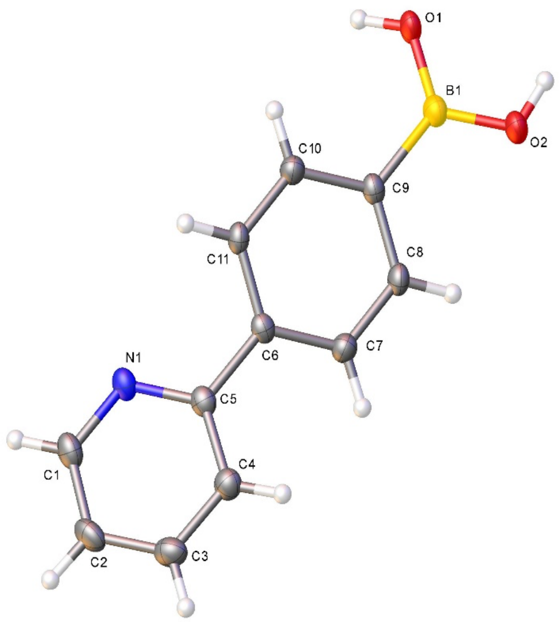

Empirical formula	$C_{11}H_{10}BNO_2$
Crystal system	monoclinic
Space group	$P2_1/n$
a/Å	14.4728(6)
b/Å	4.1308(3)
c/Å	16.3084(9)
α/°	90
β/°	93.323(5)
γ/°	90
Volume/Å^3	973.35(10)
Z	4
ρ_{calc}g/cm^3	1.358
F(000)	416.0
2Θ range /°	7.942 to 149.62
Goodness-of-fit on F^2	1.039
Final R indexes [I>=2σ (I)]	R_1 = 0.0372, wR_2 = 0.0960
Final R indexes [all data]	R_1 = 0.0441, wR_2 = 0.1021

Figure S1. Crystal structure (left) with crystal data and structure refinement for **ppy-4-BOH.**

Table S1. Summary of experimentally obtained photophysical data for Ir(III) complexes in aqueous ([PBS]=0.01 M, pH 7.4 with 2% CH_3CN and acetonitrile solutions. Estimated errors for emission wavelengths at ± 1 nm, emission lifetimes ± 10%, Φ emission quantum yields ± 20%.

		absorption	emission				
	Solvent	λ/nm (ε / 10^4 M^{-1} cm^{-1})	λ_{max} /nm	Φ % aerated		τ/ ns	
				aerated	deaerated	aerated	deaerated
**Ir-4-BOH**	CH_3CN	261(4.12), 291 sh(2.70), 307 sh(2.22), 349 sh(0.71), 415 sh(0.34)	610	4.2	11.8	55	320
	H_2O	261(4.32), 292 sh(2.70), 310 sh(2.09), 350 sh(0.69), 400 sh(0.38)	618	0.4	1.4	40	45
**Ir-ppy**	CH_3CN	254(4.48), 265 (4.33), 310 sh(1.98), 375 sh(0.56), 410 sh(0.33)	606	4.9	16.5	65	320
	H_2O	254 (4.31), 310 (1.86), 375 sh(0.52), 405 sh(0.33)	618	2.2	3.5	40	45

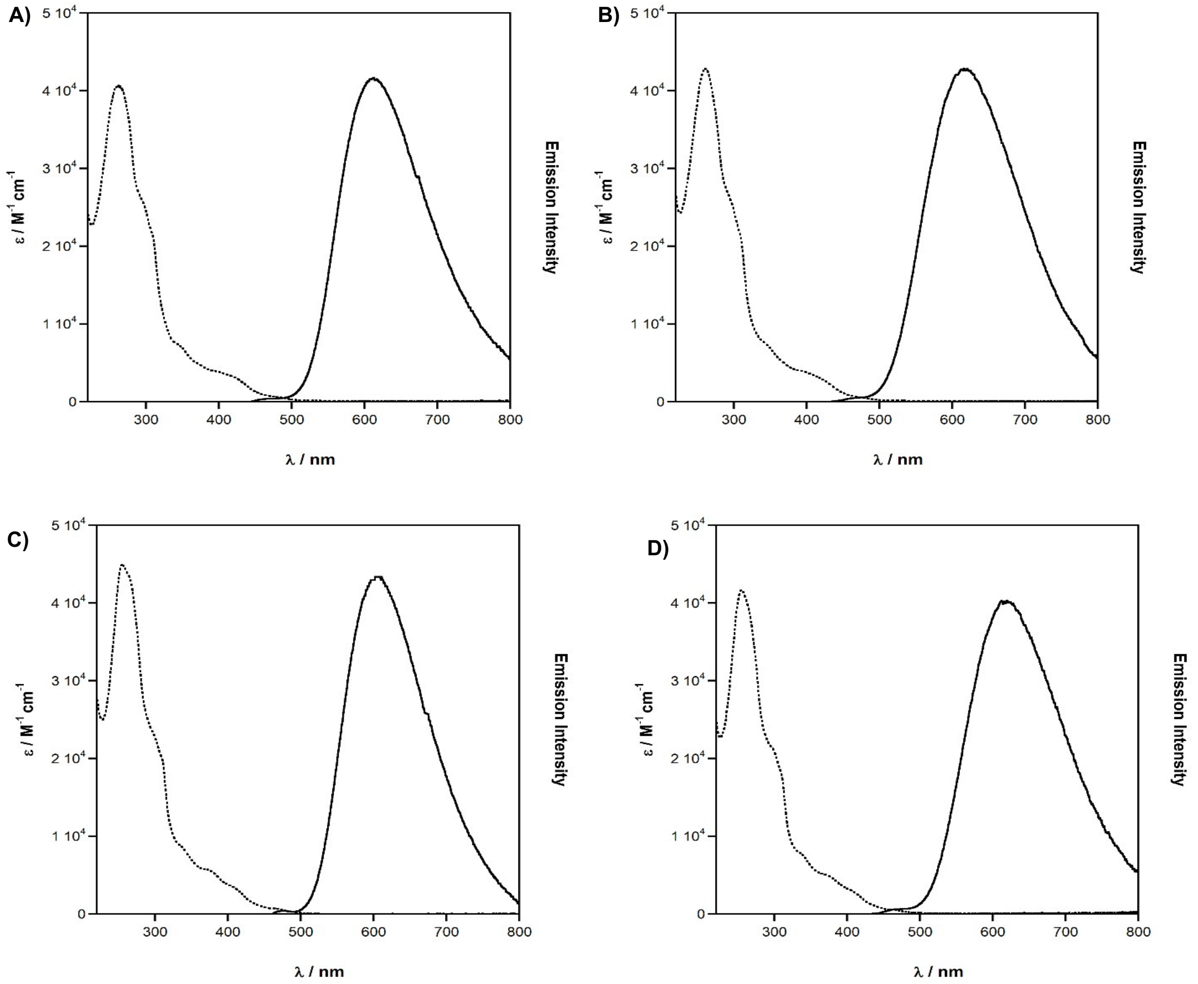

Figure S2. Electronic absorption spectra (dotted line) and emission spectra (solid line) of A) **Ir-4-BOH** in CH_3CN B) **Ir-4-BOH** in aqueous PBS (0.01 M) containing 2% CH_3CN C) **Ir-ppy** in CH_3CN D) **Ir-ppy** in aqueous PBS (0.01 M) containing 2% CH_3CN.

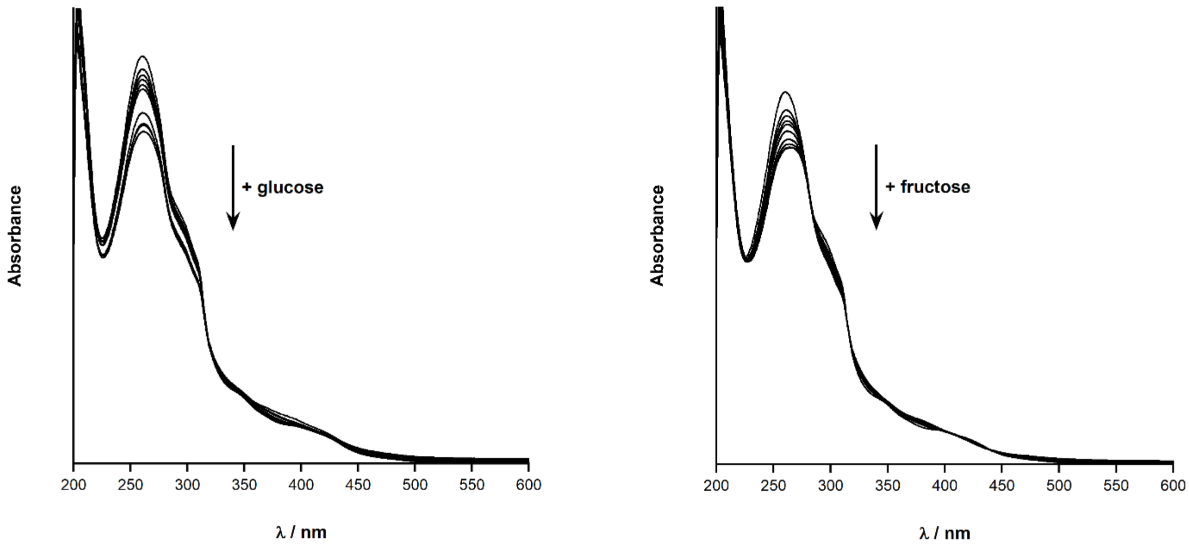

Figure S3. UV-Visible absorbance spectra of **Ir-4-BOH** (40 µM in aqueous PBS (0.01 M) with 2% CH_3CN, pH 7.4) upon additions of glucose or fructose. Monosaccharide concentrations: 0, 2, 4, 6, 8, 10, 20, 30, 40 and 50 mM.

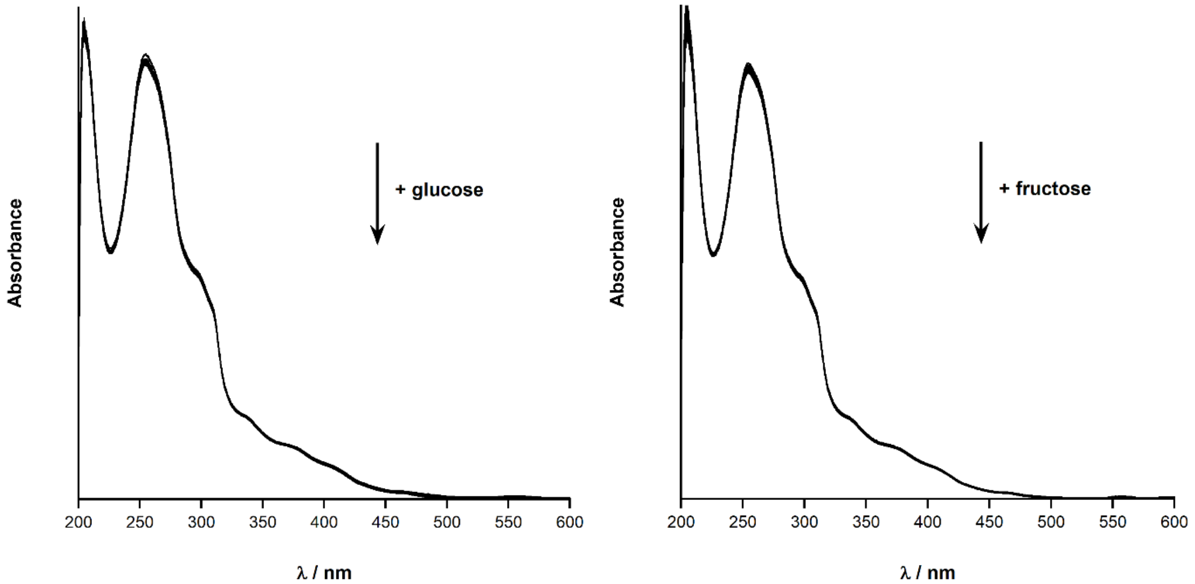

Figure S4. UV-Visible absorbance spectra of **Ir-ppy** (40 μM in aqueous PBS (0.01 M) with 2% CH_3CN, pH 7.4) upon additions of glucose or fructose. Monosaccharide concentrations: 0, 2, 4, 6, 10, 15, 20, 25 and 30 mM.

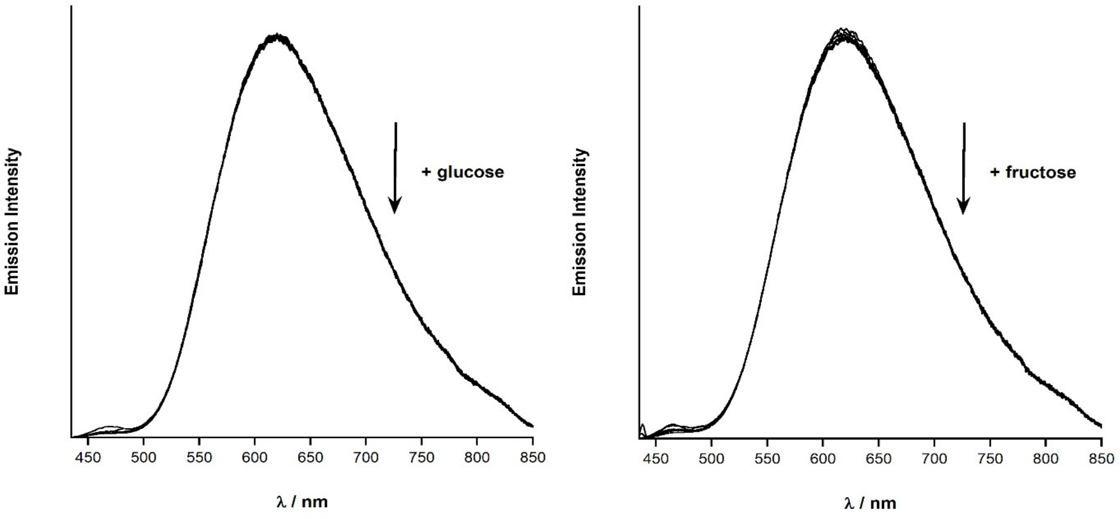

Figure S5. Emission spectra of **Ir-ppy** (40 μM in aqueous PBS (0.01 M) with 2% CH_3CN) with varying amounts of monosaccharide at concentrations of 0, 6, 10, 15, 20, 25 and 30 mM. (λ_{exc} = 350 nm).

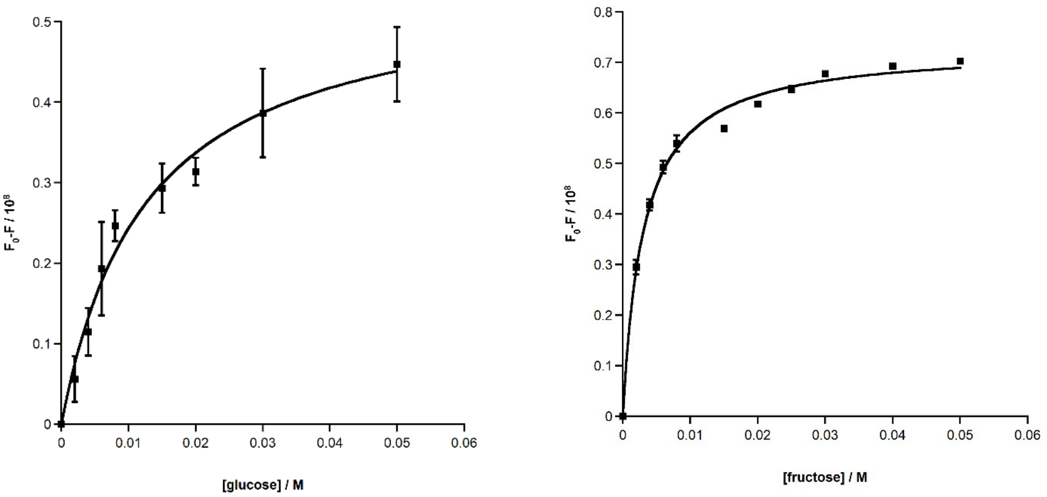

Figure S6. Nonlinear least squares fit of the binding of **Ir-4-BOH** to each monosaccharide in solution (40 μM, aq. PBS (0.01 M) with 2% CH_3CN, pH 7.4). n=3.

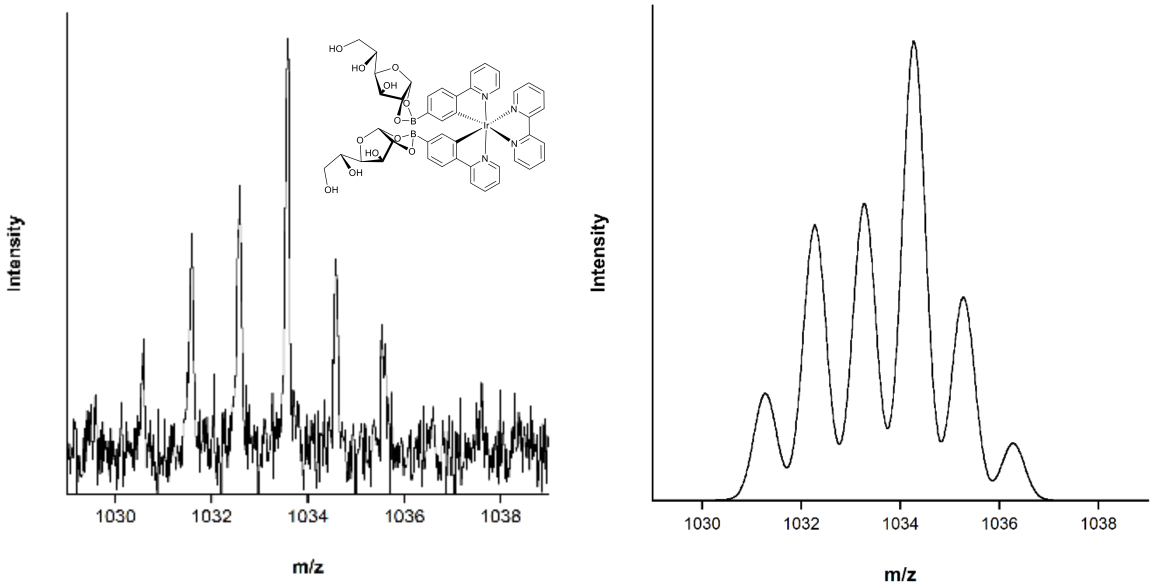

Figure S7. (Left) MALDI mass spectra for the **Ir-4-BOH•**(glucose)$_2$ species. The proposed structure of this species is displayed in the inset, (right) theoretically modelled isotope pattern for the **Ir-4-BOH•**(glucose)$_2$ species.

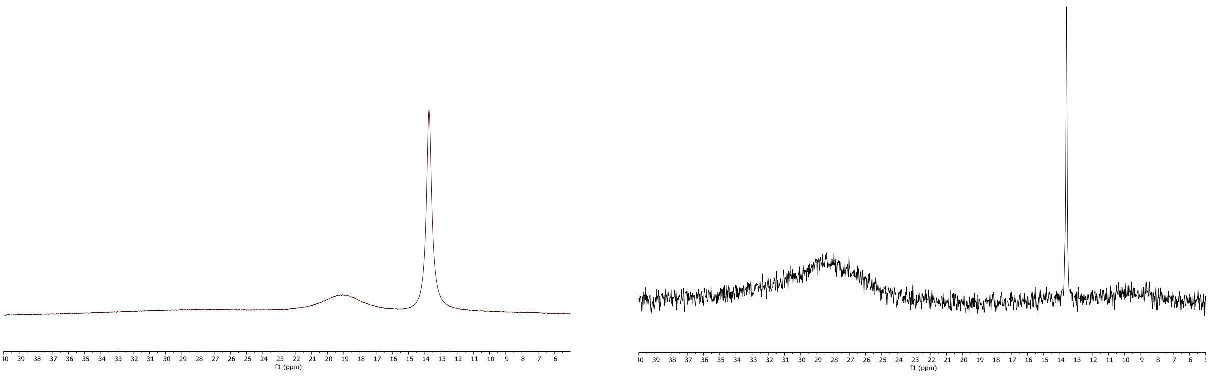

Figure S8. (left) ^{11}B NMR spectrum of the ligand **ppy-4-BOH** (left) in acetonitrile-d_3 (~30 mM) after addition of 200 mM of 3-fluorocatechol. (right) ^{11}B NMR spectrum of **Ir-4-BOH** (right) in acetonitrile-d_3 (~3mM) after addition of 100 mM 3-fluorocatechol.

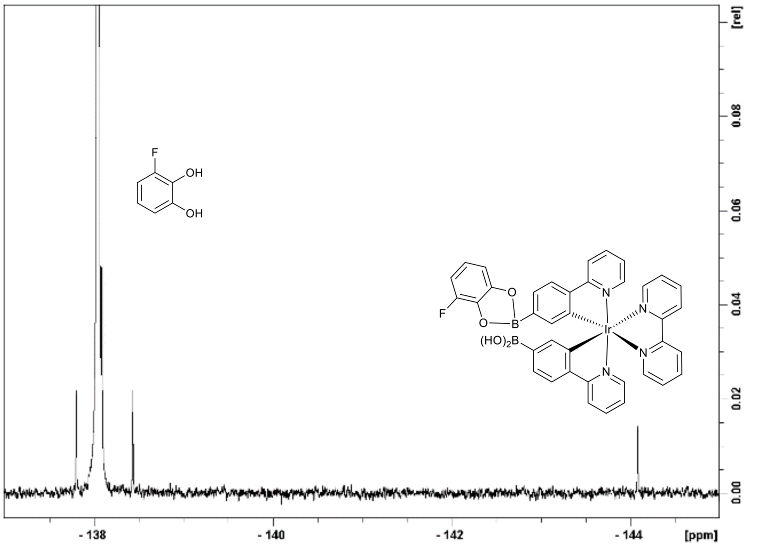

Figure S9. ^{19}F NMR spectrum of **Ir-4-BOH** in acetonitrile-d_3 (~3 mM) after addition of 100 mM 3-fluorocatechol.

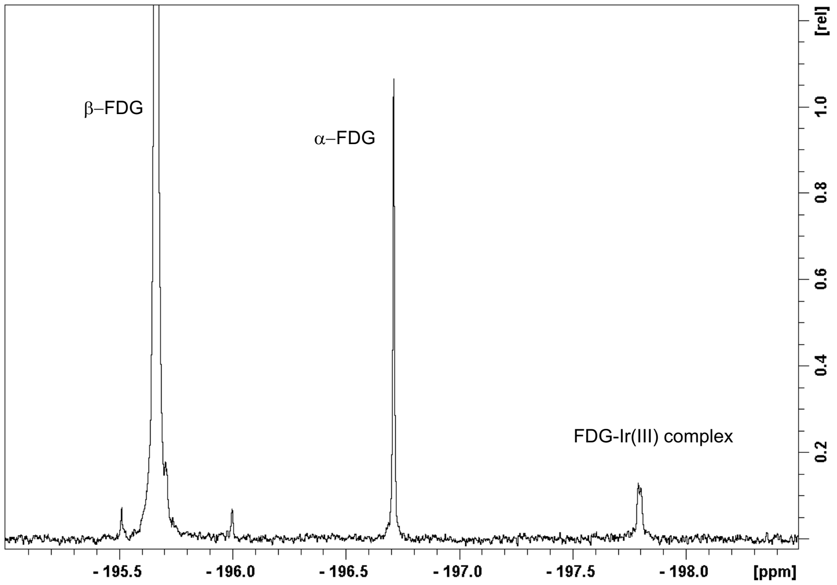

Figure S10. ^{19}F NMR spectrum of 50 mM 2-fluoro-2-deoxy-D-glucose in DMSO-d_6, after addition of 3 mM of **Ir-4-BOH**.

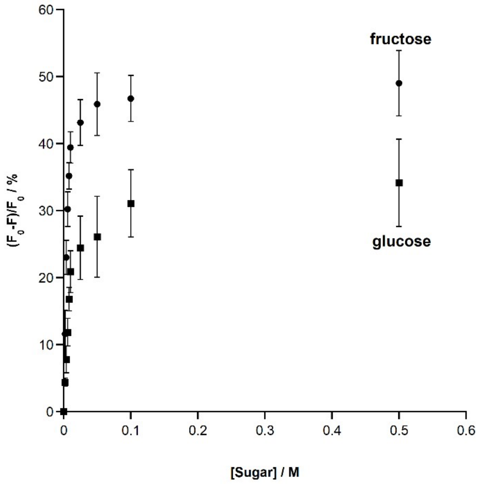

Figure S11. Binding curves for glucose and fructose (dissolved in PBS) with **Ir-4-BOH** loaded hydrogels shown as a percentage decrease of luminescence intensity by area, as a function of concentration. (λ_{exc} = 350 nm).

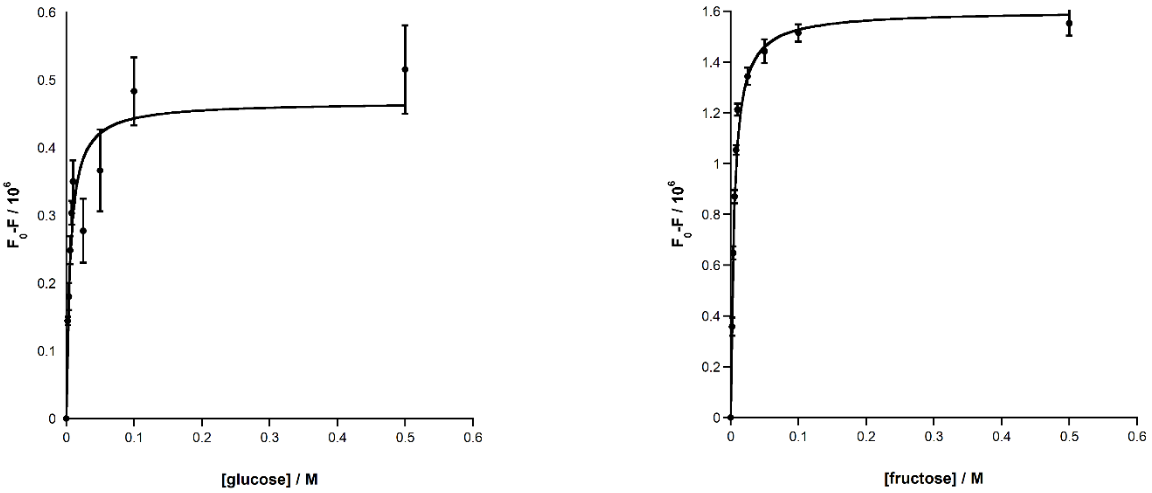

Figure S12. Nonlinear least squares fit of the binding of Ir-4-BOH hydrogels, to each sugar in solution (0.01 M aqueous PBS, pH 7.4).

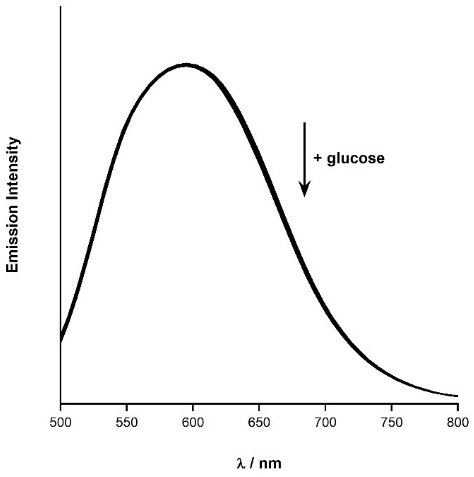

Figure S13. Emission spectra of hydrogel devices loaded with **Ir-ppy**, upon addition of with glucose. Concentrations of glucose shown are 0, 2, 4, 6, 8, 10, 25, 50 mM (λ_{exc} = 450 nm).

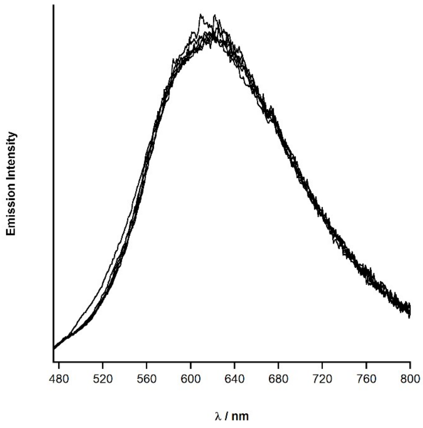

Figure S14. Luminescence spectra of hydrogel device loaded with **Ir-4-BOH** (λ_{exc} = 450 nm), taken over a 48-hour period. Excitation counts were monitored and matched at all measured points. Timepoints displayed are 5 min, 10 min, 30 min, 1.5 h, and 48 h.

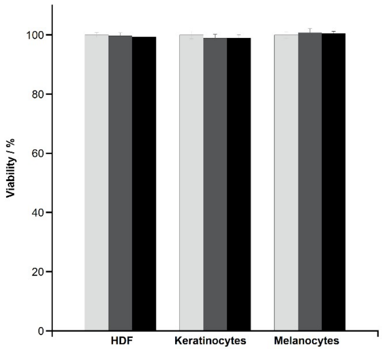

Figure S15. Cell viability of differing cell lines after 72 h incubation with non-loaded hydrogel (light grey), hydrogel loaded with **Ir-ppy** (dark grey), and hydrogel loaded with **Ir-4-BOH** (black). n = 5.

Table S2: Cellular uptake and IC_{50} values of **Ir-4-BOH** toward HeLa cells upon incubation in a sugar-free medium, and a medium containing 50 mM glucose or fructose at 37°C.

Media	Amount of iridium per HeLa cell/fmol[a]	IC_{50}/μM[b]
sugar-free	5.94 ± 0.22	8.0 ± 0.2
+glucose	2.97 ± 0.12	34.7 ± 2.5
+fructose	2.89 ± 0.13	19.1 ± 1.6

[a] Concentration of iridium associated with an average HeLa cell upon incubation with the complex (10 μM) at 37°C for 2 h, as determined by ICP-OES. [b] HeLa cells, incubation with growth medium for 24 h.

NMR Spectra

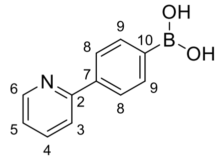

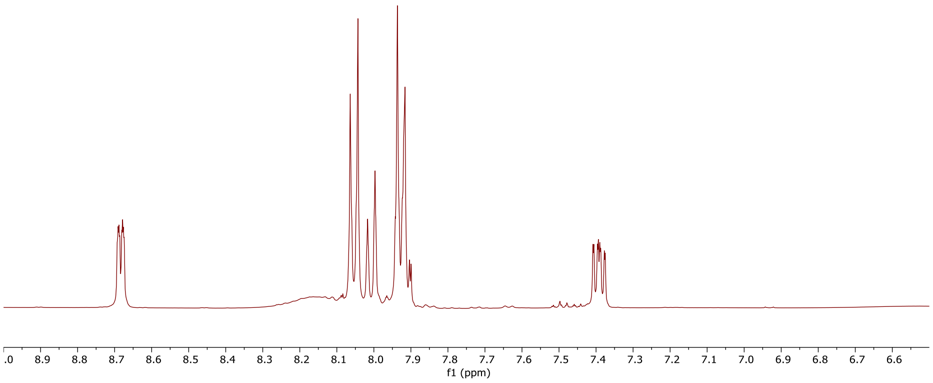

Figure S16. ^{1}H NMR spectrum of **ppy-4-BOH** with structure in DMSO-d_6 at 400 MHz.

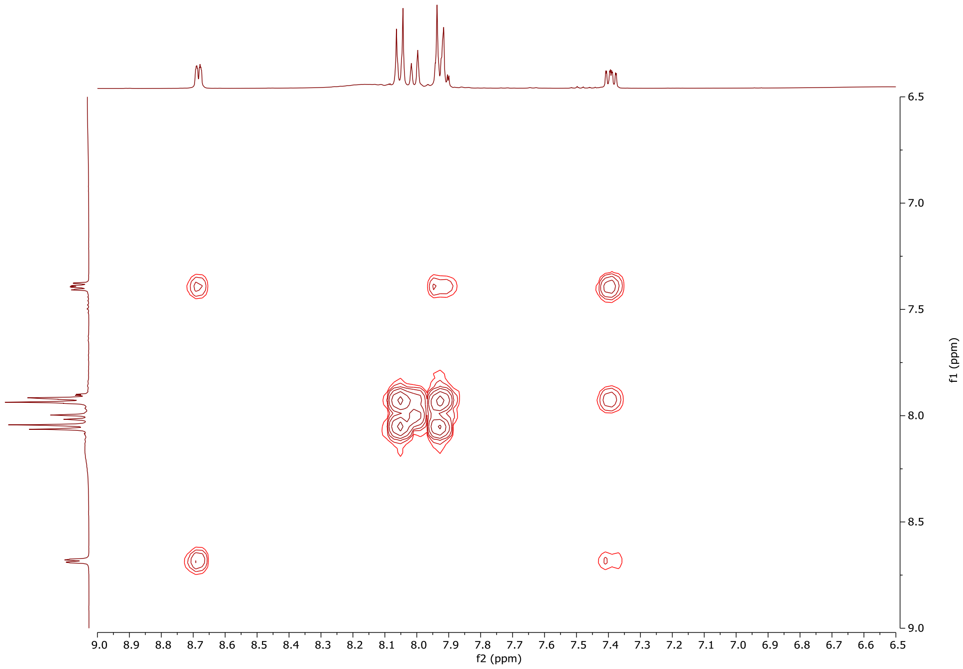

Figure S17. 2D ^{1}H-^{1}H COSY NMR spectrum of **ppy-4-BOH** in DMSO-d_6 at 400 MHz.

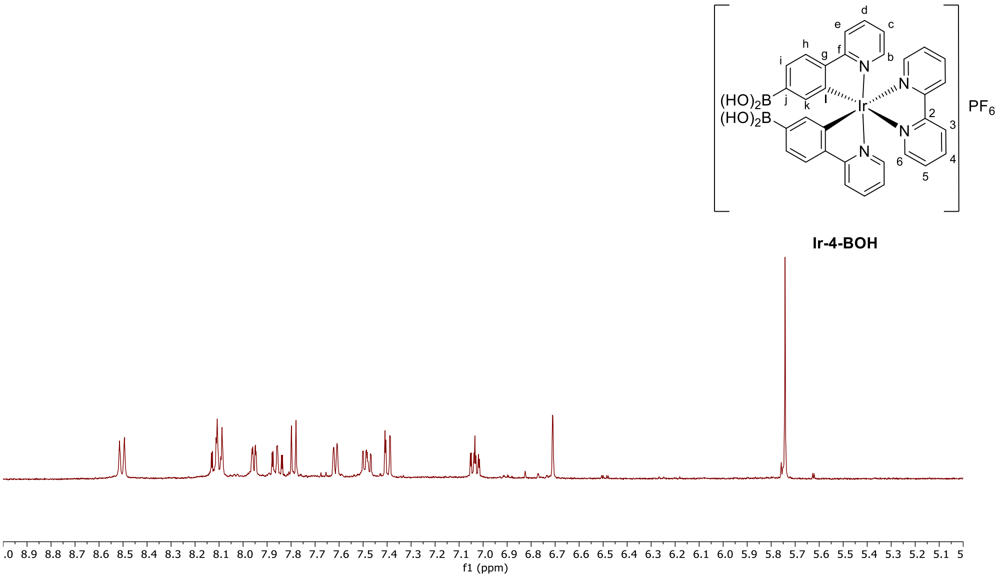

Figure S18. 1D ^{1}H NMR spectrum of **Ir-4-BOH** with structure in CD_3CN at 400 MHz.

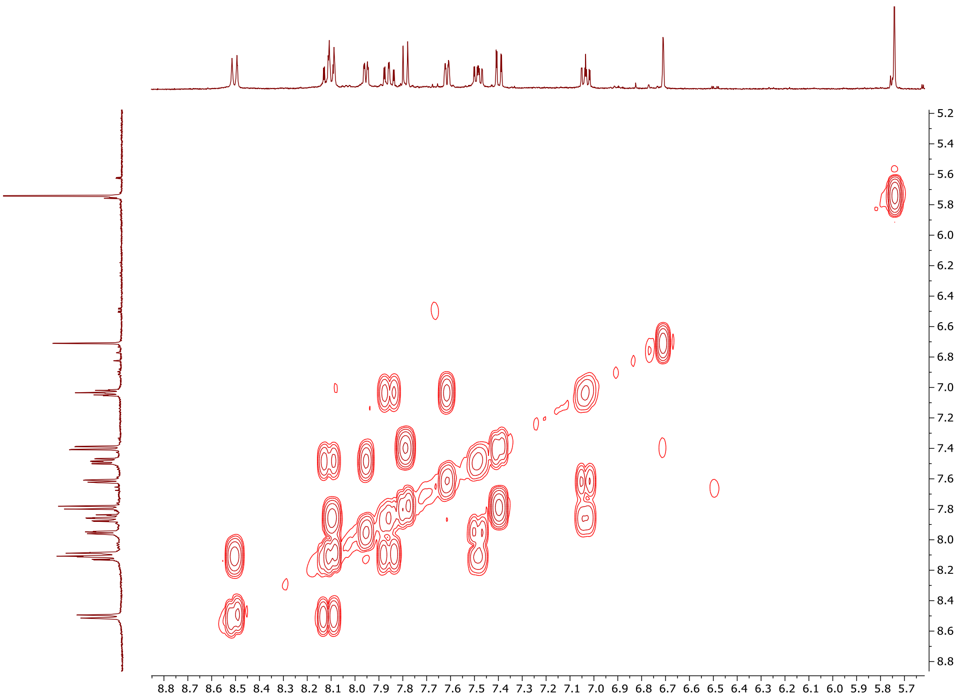

Figure S19. 2D ^{1}H-^{1}H COSY NMR spectrum of **Ir-4-BOH** in CD_3CN at 400 MHz.

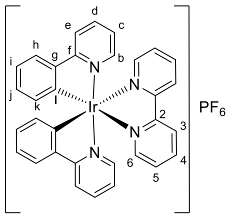

Ir-ppy

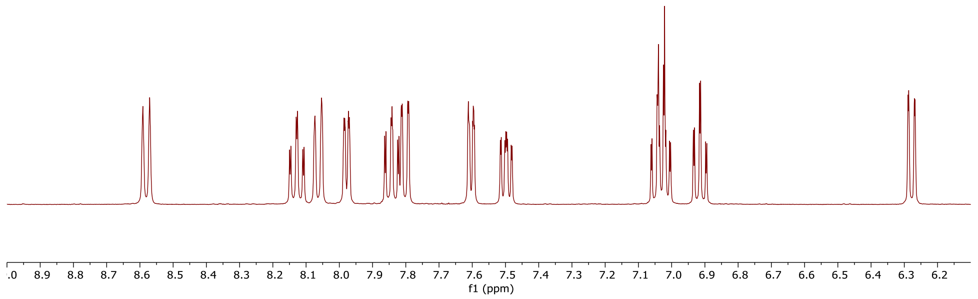

Figure S20. ^{1}H NMR spectrum of **Ir-ppy** with structure in CD_3CN at 400 MHz.

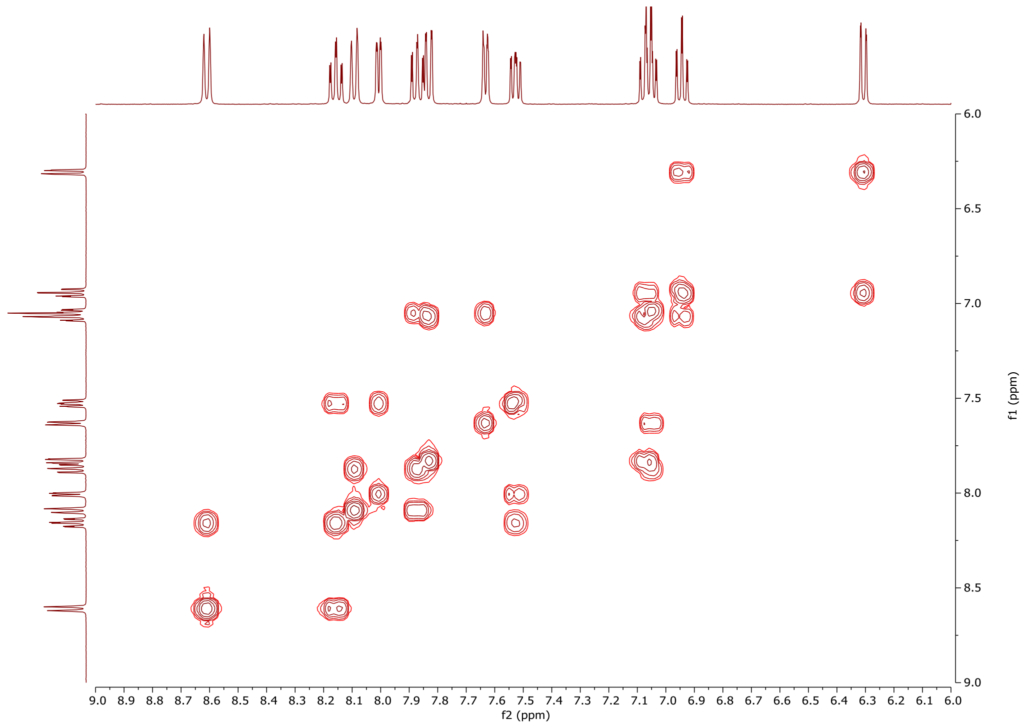

Figure S21. 2D ^{1}H-^{1}H COSY NMR spectrum of **Ir-ppy** in CD_3CN.

Experimental Section

General Considerations

Starting materials and solvents were obtained from Sigma-Aldrich, Fluka, Fisher Scientific, or Acros Chemicals and used without any further purification. D-(-)-fructose was purchased from Scientific Laboratory Supplies, and the □-D-glucose from Sigma. Flash column chromatography was performed using LC60A 40−63 μm silica gel (Merck) or activated, neutral Brockmann I grade alumina (Merck). All reactions were performed under an argon atmosphere unless stated otherwise. NMR spectra were measured on a Bruker AVANCE NEO spectrometer operating at 400 MHz for ^{1}H and fitted with a BBFO "smart" probe) or a Varian spectrometer operating at 400 MHz for ^{1}H. ^{1}H and ^{13}C assignments were confirmed by 2D ^{1}H-^{1}H COSY, 2D ^{1}H-^{13}C HSQC and 2D ^{1}H-^{13}C. The ^{11}B NMR spectra were processed by running a sample of blank solvent with matching conditions and subtracting the signal from the ^{11}B spectra of the samples containing boronic acid species to eliminate background signal. Electrospray mass spectrometry was carried out on a Waters Micromass Q-TOF, and MALDI mass spectrometry on a Bruker Flextreme MALDI-TOF. UV−Visible spectra were collected using 1 cm path length polished quartz cuvettes on an Agilent Cary 60 spectrophotometer. Luminescence spectra for saccharide binding studies were carried out using a Photon Technology International luminescence spectrometer with a 150 W xenon arc lamp as the excitation source. Felix analysis software was used to record data, and all spectra were corrected for photomultiplier tube and instrument response. Other steady state luminescence measurements, quantum yields, and time resolved studies were recorded on an Edinburgh Instruments FLS920 steady state and time-resolved spectrometer. The detection system used an incorporated R928 Hamamatsu photomultiplier tube. The emission monochromator is fitted with gratings blazed at 500 nm. F900 spectrometer analysis software was used to record the data, and all spectra were corrected for photomultiplier and instrument response. Luminescent lifetimes were recorded with EPL-375 laser as an excitation source and fitted using Edinburgh Instruments FAST software with estimated error of ± 10%. Argon was used to degas samples by bubbling through the solution inside a cuvette fitted with a tight sealed septum for 15 min. Quantum yield measurements were taken using an integrating sphere attachment supplied by Edinburgh Instruments. Saccharide Binding by Photoluminescence Photoluminescence experiments for saccharide binding were carried out in 0.01 M aqueous PBS containing 2 % acetonitrile. Sugars were first added as a solution in PBS. 2.5 □L aliquots were added up to a concentration of 10 mM, after which saccharides were added as powders, to avoid dilution effects. Samples for mass spectrometry were prepared by first making a saturated solution of Ir(III) complex in a 0.1 mL aqueous solution (1:1, MeCN:H_2O) and adding 1.8 mg (0.01 mol) of glucose. Electrochemistry Cyclic Voltammograms were recorded using a potentiostat workstation (CH Instrument model 760E, CH Instruments, Austin, TX) using a conventional three-electrode configuration cell. A glassy carbon electrode (IJ Cambria, UK) of 3 mm diameter (area 0.07 cm^2) was used as the working electrode and a platinum wire as a counter electrode. All potentials were quoted versus an Ag/AgCl reference electrode and all the measurements were recorded at room temperature. ECL measurements were performed using a Hamamatsu power supply photosensor modules with an open window of 530 V coupled with the potentiostat. The ECL signal was acquired using a PMT and the optical fiber model supplied by Hamamatsu positioned perpendicularly to the GCE. CV and ECL curves were recorded at scan rate of 50 mVs^{-1}. Hydrogel Studies Hydrogel devices were fabricated using an AnalytikJena UVP CL-1000 crosslinker, programmed to 999,900 $\mu J/cm^2$ and irradiation time of 1 min. A Thorlabs CPS450) laser was used as an excitation source, with energy of 3 J/cm^2 pulsed for 3 seconds. An Ocean Optics USB 2000 was used as the detector, with bifurcated fibre optical fibres. Measurements were taken in a cuvette with parallel clear sides in dark conditions. To investigate potential toxicity of the hydrogels to cells in the skin, primary adult human epidermal keratinocytes (Keratinocytes; ThermoFisher Scientific, cat no. C0055C), primary adult human epidermal melanocytes (lightly pigmented donor) (Melanocytes; ThermoFisher, cat no. C0245C) and primary adult human dermal fibroblasts (HDF; ThermoFisher, cat no. C0135C) were expanded in Epilife CF (ThermoFisher, Cat no. MEPICF500), human melanocyte growth supplement (ThermoFisher; Cat no. S0025) and DMEM supplemented with 10% FCS (ThermoFisher), respectively. Cells were used between passage 7-10 for all experiments. The MTT assay was then used to assess toxicity of gels to human skin cells, as described by us previously (Badaway et al., 2018). Briefly, 5 × 10^3 cells of each population were plated in six-well plates (n = 5 wells/condition), covered with 2 mL of each respective growth medium, defined masses of gels were immersed into the culture medium and left in contact with cells for 72 h at 37 °C and 5% CO2. After 72 h, supernatants were removed and cells were washed in PBS and the MTT assay was performed according to the manufacturer's instructions (R&D Systems, Watford, UK). The resulting absorbances were read at 570 nm and absorbance units calculated from triplicate readings after subtraction of blank wells (culture media only).

Cell cultures HeLa cells were grown in Dulbecco's Modified Eagle Medium (DMEM) supplemented with 10% fetal bovine serum (FBS) and 1% penicillin/streptomycin at 37 °C under a 5% CO_2 atmosphere. They were subcultured every 2 – 3 days.

ICP-OES measurements HeLa cells were grown in three 35-mm tissue culture dishes and incubated at 37 °C under a 5% CO_2 atmosphere. After 48 h incubation, the growth medium was replaced by a sugar-free medium or a medium supplemented with glucose (50 mM) or fructose (50 mM) containing Ir-4-BOH (10 μM) in growth medium/DMSO (99:1, *v*/*v*), and incubated at 37 °C under a 5% CO_2 atmosphere. After 2 h, the medium was removed, and the cell layer was washed gently with PBS (1 mL × 3). The cells were trypsinized and harvested with PBS (2 mL). The resultant solution was heated with 65% HNO_3 (2 mL) at 70 °C for 2 h, cooled to room temperature, and analyzed using an Optima 8000 ICP-OES system (PerkinElmer, Inc., USA).

Dark cytotoxicity assays HeLa cells were seeded in three 96-well flat-bottomed microplates (*ca.* 10 000 cells per well) in a growth medium (100 μL) and grown at 37 °C under a 5% CO_2 atmosphere. After 24 h incubation, the growth medium was replaced by a sugar-free medium or a medium supplemented with glucose (50 mM) or fructose (50 mM) containing Ir-4-BOH at concentrations ranging from 10^{-4} to 10^{-7} M in growth medium/DMSO (99:1, *v*/*v*). Wells containing untreated cells were used as blank controls. The microplates were incubated at 37 °C under a 5% CO_2 atmosphere for 24 h. Then, MTT in PBS (10 μL, 5 mg mL^{-1}) was added to each well and the microplates were incubated at 37 °C under a 5% CO_2 atmosphere for 4 h. The growth medium was then removed, and DMSO (200 μL) was added to each well. The microplates were further incubated at 37 °C for 15 min. The absorbance of the solutions at 570 nm was measured with a Powerwave XS MQX200R microplate spectrophotometer (BioTek Instruments Inc., Winooski, VT). The IC_{50} values of the complexes were determined from dose dependence of surviving cells after exposure to the complexes.

Live-cell confocal imaging HeLa cells in growth medium were seeded on sterilized coverslip in three 35-mm tissue culture dishes and grown at 37 °C under a 5% CO_2 atmosphere. After 48 h incubation, the growth medium was replaced by a sugar-free medium or a medium supplemented with glucose (50 mM) or fructose (50 mM) containing Ir-4-BOH (10 μM) in medium/DMSO (99:1, *v*/*v*), and incubated at 37 °C under a 5% CO_2 atmosphere for 2 h. The growth medium was removed, and the cell layer was washed gently with PBS (1 mL × 3). After that, the coverslip was mounted onto a sterilized glass slide and imaged using a Leica TCS SPE (inverted configuration) confocal microscope with a 63 × oil-immersion objective and an excitation wavelength at 405 nm.

Experimental Section

Synthesis of ppy-4-BOH To a stirring mixture of Mg (610 mg, 25.0 mmol) and LiCl (25.0 mL, 0.5 M in THF, 12.5 mmol), diisobutylaluminium hydride (0.1 mL, 1 M in toluene, 0.1 mmol) was added, and the solution stirred for 5 min. After this time 2-(4-bromophenyl)pyridine (2.34 g, 10.0 mmol) was added at room temperature, and stirring continued for 1 h. The mixture was cooled to 0 °C and the trimethyl borate (2.09 mL, 20.0 mmol) was added and stirred for 5 min, before quenching with HCl (0.1 M, 5 mL). The reaction mixture was extracted with EtOAc (2 x 20 mL) and the organic layer was isolated and dried over Na_2SO_4 and the solvent evaporated in vacuo to give a crude solid that was recrystallised (CH_3CN:H_2O, 1:1) to yield a white crystalline solid. (1.68 g, 8.44 mmol, 84%) ^1H NMR (400 MHz, DMSO-d_6) δ 8.68 (ddd, J = 4.8, 1.1, 0.9 Hz, 1H, H-6), 8.17 (m, 2H, $B(OH)_2$), 8.05 (d, J = 8.2 Hz, 2H, H-8), 8.01 (dt, J = 8.0, 1.1 Hz, 1H, H-4), 7.97 – 7.89 (m, 3H, H-3, H-9), 7.39 (ddd, J = 8.0, 4.9, 1.2 Hz, 1H, H-5). ^{13}C NMR (101 MHz, DMSO-d_6) δ 156.2 (C-7), 149.6 (C-6), 140.0 (C-2), 138.2 (C-3), 135.5 (C-10), 135.0 (C-9), 126.0 (C-8), 123.3 (C-5), 121.2 (C-4). m/z (ESI-TOF) 200.22 $[M+H]^+$.

Synthesis of Ir-4-BOH ppy-4-BOH (60 mg, 0.30 mmol) and $IrCl_3.H_2O$ (50 mg, 0.16 mmol) were dissolved in 2-ethoxyethanol (6 mL) and H_2O (2 mL). The solution was stirred at 100 °C for 24 h, cooled, and the resulting solution was evaporated to dryness. The residue was washed with H_2O (2 x 10 mL) and hexane (30 mL) to yield the Ir(III) dimer. The dimer (30 mg, 0.024 mmol) and 2,2'-bipyridine (8.5 mg, 0.05 mmol) were added to dry dichloromethane and methanol (1:3, 10 mL). The solution was heated to 60 °C and stirring continued at this temperature for 6 h. The solution was filtered and the volume reduced in vacuo to 1 mL; 2 mL of a methanolic solution of NH_4PF_6 (1g, 6.1 mmol) were added to yield a precipitate. The product was purified by column chromatography (silica gel, 9:1, DCM:MeOH) before drying in vacuo to yield a yellow crystalline solid (13 mg, 0.015 mmol, 30 %). ^1H NMR (400 MHz, CD_3CN) δ 8.51 (d, J = 8.1 Hz, 2H, H-3), 8.13 – 8.08 (m, 4H, H-4,e), 7.95 (ddd, J = 5.5, 1.7, 0.8 Hz, 2H, H-6), 7.86 (ddd, J = 8.2, 7.3, 1.5 Hz, 2H, H-d), 7.79 (d, J = 7.5 Hz, 2H, H-h), 7.62 (ddd, J = 5.9, 1.6, 0.8 Hz, 2H, H-b), 7.48 (ddd, J = 7.7, 5.5, 1.3 Hz, 2H, H-5), 7.40 (dd, J = 7.5, 1.2 Hz, 2H, H-i), 7.04 (ddd, J = 7.3, 5.9, 1.5 Hz, 2H, H-c), 6.71 (d, J = 1.2 Hz, 2H, H-k), 5.74 (s, 4H, BOH). ^{11}B NMR (128 MHz, CD_3CN) δ 28. λ_{max} (CH_3CN)/nm (ε/dm^3 mol^{-1} cm^{-1}) 261 (41200), 291 (27000), 307 (22200), 349 (7100), 415 (3400). HRMS (ESI): m/z calcd for $C_{32}H_{26}B_2N_4O_4Ir^+$: 745.1768 $[M-PF_6]^+$; Found: 745.1769.

Synthesis of Ir-ppy The synthetic method of Ohsawa et al. was followed,[1] to give Ir-ppy (32 mg, 0.04 mmol, 68 %). ^1H (400 MHz, CD_3CN) δ 8.58 (d, J = 8.0, 2H, H-3), 8.13 (td, J = 8.0, 1.6 Hz, 2H, H-4), 8.06 (d, J = 8.0 Hz, 2H, H-e), 7.98 (ddd, J = 5.5, 1.6, 0.7 Hz, 2H, H-6), 7.83 (ddd, J = 8.0, 7.0, 1.6 Hz, 2H, H-d) 7.80 (dd, J = 7.6, 1.3 Hz, 2H, H-h), 7.60 (ddd, J = 5.8, 1.6, 0.7 Hz, 2H, H-b), 7.50 (ddd, J = 8.0, 5.4, 1.1 Hz, 2H, H-5), 7.09 – 6.99 (m, 4H, H-c, H-j), 6.91 (td, J = 7.6, 1.4 Hz, 2H, H-i), 6.28 (dd, J = 7.5, 0.8 Hz, 2H, H-k) ^{13}C NMR (101 MHz, CD_3CN) δ 168.3 (C-f), 156.8 (C-2), 151.5 (C-6), 151.3 (C-l), 150.1 (C-b), 145.0 (C-g), 140.3 (C-4), 139.5 (C-d), 132.4 (C-h), 131.3 (C-i), 129.3 (C-5), 125.8 (C-k), 125.7 (C-3), 124.4 (C-c), 123.5 (C-j), 120.8 (C-e). λ_{max} (CH_3CN)/nm (ε/dm^3 mol^{-1} cm^{-1}) 254(44800), 265 (43300), 310 sh(19800), 375 sh(5600), 410 sh(3300). m/z (ESI) 657.17 $[M-PF_6]^+$.

Preparation of Ir(III) Doped Hydrogel 2-Hydroxyethyl methacrylate (900 mg, 6.9 mmol), di(ethyleneglycol) dimethacrylate (50 mg, 0.3 mmol), 2-hydroxy-2-methylpropiophenone (20 mg, 0.1 mmol) were added to 1mL of DMSO, to which was added 50 μL of a 3.5 mM (0.2 μmol) solution of Ir-4-BOH or Ir-ppy in DMSO. The solution was stirred for 5 min and then placed under a UV-crosslinker for 1 min. Once set, the gel was cut out of the mould and transferred to a UV transparent cuvette.

Crystal structure determination of ppy-4-BOH A suitable crystal was selected and a dataset for ppy-4-BOH was measured on an Agilent SuperNova diffractometer using an Atlas detector. The data collection was driven and processed and an absorption correction was applied using CrysAlisPro. The structure was solved using ShelXT,[2] and was refined by a full-matrix least-squares procedure on F2 in ShelXL.[2] Figures and reports were produced

using OLEX2. All non-hydrogen atoms were refined with anisotropic displacement parameters. The hydrogen atoms bonded to O(1) and O(2) were located in the electron density and freely refined. All remaining hydrogen atoms were fixed as riding models and the isotropic thermal parameters (Uiso) were based on the Ueq of the parent atoms.

References

[1] Y. Ohsawa, S. Sprouse, K. A. King, M. K. DeArmond, K. W. Hanck and R. J. Watts, *J. Phys. Chem.* **1987**, *91*, 1047-1054.

[2] a) G. M. Sheldrick, *Acta Cryst.* **2015**, *A71*, 3-8; b) G. M. Sheldrick, *Acta Cryst.* **2015**, *C71*, 3-8; c) O. V. Dolomanov, L. J. Bourhis, R. J. Gildea, J. A. K. Howard and H. Puschmann, *J. Appl. Crystallogr.* **2009**, *42*, 339-341.
